# Congenital hemophilia A diagnosed with postoperative hemorrhage after thyroidectomy for papillary thyroid carcinoma: a case report

**DOI:** 10.1186/s40792-021-01272-x

**Published:** 2021-08-21

**Authors:** Marie Saitou, Muneo Okamoto, Ryuta Nagaoka, Tomoo Jikuzono, Masaomi Sen, Hiroko Kazusaka, Mami Matsui, Iwao Sugitani

**Affiliations:** Nippon Medical School, 1-1-5 Bunkyo-ku, Sendagi, Tokyo, Japan

**Keywords:** Thyroidectomy, Postoperative hemorrhage, Papillary thyroid carcinoma, Hemophilia A

## Abstract

**Background:**

Postoperative bleeding in thyroid surgery is a serious complication with fatal outcomes. Risk factors for postoperative hemorrhage have been reported as old age, male sex, Graves' disease, use of anticoagulants, and hematological disorders. Among the hematological diseases, congenital hemophilia is an inherited bleeding disorder characterized by absence or reduced levels of clotting factors VIII or IX. Most patients with hemophilia display bleeding symptoms during infancy or childhood, but diagnosis could be delayed in mild cases. We report a case of congenital hemophilia A that was diagnosed after three episodes of postoperative bleeding after thyroid surgery.

**Case presentation:**

A 46-year-old man developed repeated postoperative hemorrhage after thyroid surgery for thyroid cancer. In this case, several irregularities were seen in the postoperative course, such as a relatively long interval between surgery and bleeding, the lack of an obvious bleeding point, fresh red blood dripping from the drain insertion site on the second postoperative day, and repeated bleeding three times. We therefore considered that the cause of postoperative hemorrhage might be other than the surgical operations. After a thorough examination, hemophilia A was diagnosed.

**Conclusions:**

Hemophilia is a risk factor for postoperative bleeding in thyroid surgery. However, mild hemophilia shows normal prothrombin time and activated partial thromboplastin time. We encountered a case of papillary thyroid carcinoma associated with congenital hemophilia A, which was diagnosed after repeated bleeding.

## Background

Postoperative hemorrhage in thyroid surgery is a life-threatening complication that can cause tracheal compression and asphyxiation. Risk factors for postoperative hemorrhage include advanced age, male sex, Graves' disease, use of anticoagulants, and complications of hematological diseases [1–5]. Among the hematological diseases, congenital hemophilia is an inherited bleeding disorder characterized by the absence or reduced levels of clotting factor VIII or IX. Most patients with hemophilia display bleeding symptoms during infancy or childhood, but diagnosis can be delayed in mild cases. We report a case of congenital hemophilia A that was diagnosed after three repeated episodes of postoperative bleeding after thyroid surgery.

## Case presentation

A 43-year-old man presented with a thyroid nodule detected on screening carotid ultrasonography. He had shown hypertension at 42 years old and he had no contributory family history, including coagulopathies. He was referred to our hospital for diagnosis and treatment of the nodule. Blood tests showed: hemoglobin, 15.1 g/dL; platelets, 27.5 × 10^4^/µL; prothrombin time (PT), 11.0%; and activated partial thromboplastin time (aPTT), 32.0 s. Coagulability was normal. Ultrasonographic findings showed an 11 × 11 × 14-mm, hypoechoic nodule with irregular margins on the left lobe of the thyroid gland. No obvious extrathyroidal extension or lymph node metastasis was identified. Fine needle aspiration biopsy revealed findings suggestive of papillary thyroid carcinoma. Computed tomography showed a nodule in the left lobe of the thyroid gland and no distant metastatic findings in the lung.

**Fig. 1:**
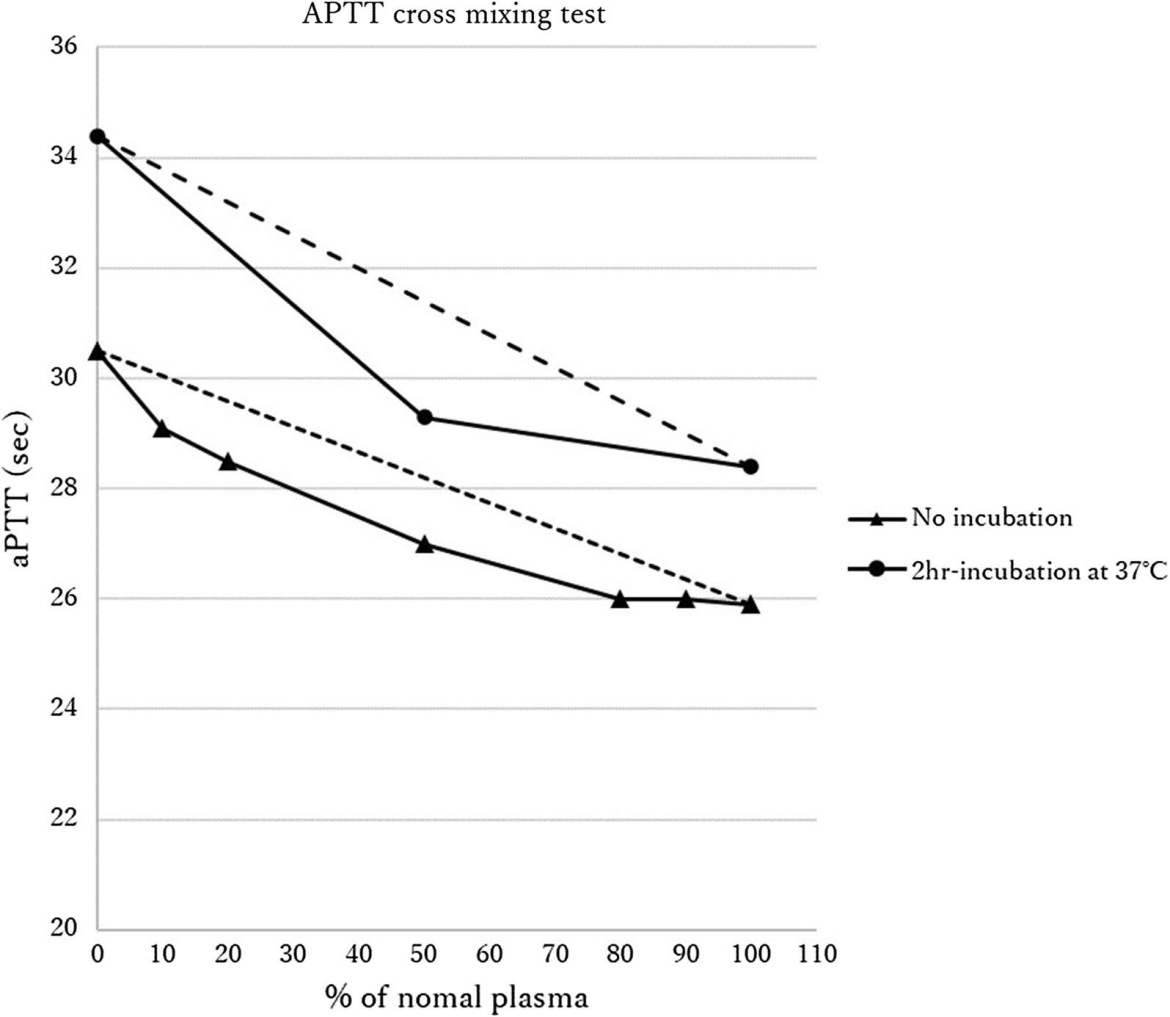
Results of mixing test. Mixing test detected inhibitor of coagulation factor. A convex upward curve indicates the presence of inhibitors, including coagulation factor-neutralizing antibodies, while a convex downward curve indicates the presence of a factor deficiency

The patient underwent left lobectomy and central compartment lymph node dissection under the diagnosis of papillary thyroid carcinoma (cT1bN0M0). The histopathological diagnosis was papillary thyroid carcinoma (pT2N1aM0). Intra-operatively, there was no finding of abnormal hemostasis and intraoperative blood loss was less than 1 ml. Operative time was 54 min. No sign of bleeding was seen on examination at 20-h postoperatively. However, the patient’s neck appeared swollen at 27 h postoperatively. Emergency reoperation was performed to evacuate hematoma. No arteriovenous points of bleeding were evident, but diffuse oozing was identified. Two days after the reoperation, on removal of the drainage tube, fresh red blood was seen dripping from the drain insertion site. We therefore applied pressure around the drain insertion site with gauze for 2 days, and prescribed carbazochrome and tranexamic acid for 3 days. The patient was discharged from hospital on postoperative day (POD) 6 after confirming that neck swelling had resolved. We had recommended continued hospitalization and observation, but the patient strongly desired to be discharged to home. On the fifth day after discharge (POD 11), neck swelling was observed again. Since the swelling was mild, the hematoma was conservatively absorbed. We considered the possibility that repeated postoperative hemorrhages were attributable to abnormalities in blood coagulation factors. Subsequent testing revealed factor VIII antibody activity had decreased to 33.9%, van Willebrand factor (vWF) was within the reference range (Table 1), and cross-mixing test showed a downward convex pattern (Fig. 1). The patient was also found to have shown difficulty in achieving hemostasis during a previous tooth extraction. He had factor VIII deficiency, normal level of vWF and bleeding episode. Eventually, congenital hemophilia A was diagnosed.

**Table 1:** The main blood test of coagulation system

		Units	Normal value
WBC	6700.0	/µL	33–86
RBC	477.0	× 10^4^/µL	435–555
HGB	14.9	g/dL	13.7–16.8
PLT	34.4	× 10^4^/µL	15.8–34.8
PT	11.2	s	
PT	1.1	%	80.0–120.0
PT-INR	1.0		
aPTT	32.1	s	24.0–39.0
FIB	336.0	mg/dL	170–400
Antithrombin III	1.2	%	80–120
TAT	1.9	µg/L	< 3.0
PIC	0.5	µg/mL	< 0.8
FDP	3.4	µg/mL	< 10.0
Plasminogen	109.0	%	80–130
Antiplasmin	115.0	%	80–130
Factor VIII activity	33.9	%	78–165
Factor IX activity	99.2	%	67–152
Factor XIII inhibitor	88.0	%	70–140
von Willebrand factor	69.0	%	50–150

The patient factor VIII antibody activity decreased to 33.9%, vWF was within the reference value

## Discussion

Congenital hemophilia A is an X-linked recessive coagulation disorder caused by factor VIII deficiency. This disorder is characterized by a bleeding tendency, a normal PT, and a prolonged aPTT. As the most common congenital coagulopathy, hemophilia A occurs in 1–2 per 10,000 male births [6]. In severe hemophilia, deep bleeding such as intra-articular and intramuscular hemorrhage is seen in infancy and early childhood due to increased mobility and activity. Unusual purpura or hematoma may also trigger the diagnosis [7]. In this case, hemophilia was not diagnosed preoperatively because the patient showed no symptoms of blood disorder. In hemophilia, the PT and platelet count remain normal and the aPTT is prolonged. Patients with mild hemophilia may have a normal aPTT. If the aPTT is prolonged, mixing studies can determine whether the patient has a factor deficiency or presence of an inhibitor. Factor activity levels are measured for the relevant factor if a familial defect is known, or for all of these factors in new cases. The diagnosis of hemophilia A requires confirmation of a factor VIII activity level below 40% of normal or, in some circumstances where the factor VIII activity level is ≥ 40%, a pathogenic factor VIII gene mutation. A normal vWF antigen should also be documented to eliminate the possibility of some forms of von Willebrand disease. In this case, factor VIII activity level of the patient was 34% and a normal level of vWF antigen, so hemophilia A was diagnosed. Patients with hemophilia A need regular replacement therapy or on-demand replacement therapy (i.e., factor given only in the setting of acute bleeding or surgery). Regular replacement therapy is expensive and places substantial burdens on the patient for recurrent venous access, but patients with mild pathology can receive on-demand therapy only. The "Guidelines for Hemostatic Treatment of Hemophilia Patients without Inhibitors" by The Japanese Society on Thrombosis and Hemostasis recommend that patients undergoing surgery under general anesthesia should be administered a trough factor level of at least 80% for 5–10 days, and thereafter a trough factor level of at least 30% for 3–5 days [8].

Postoperative hemorrhage in thyroid surgery can cause laryngeal edema, which can lead to asphyxia. In the case of postoperative bleeding, emergency reoperation to evacuate hematoma is mandatory. In our case, reoperation was performed immediately after the bleeding was detected. However, some points of difference from the usual progress of postoperative bleeding were apparent. First, the onset of postoperative bleeding was slower than usual. One report found that 95.7% of postoperative bleeding occurs within 24 h after surgery [9], but our patient experienced bleeding after 27 h. Second, no arteriovenous vessels were detected as bleeding points. In general, postoperative hemorrhage from thyroid surgery occurs in 1–1.5% [4]. According to a previous report [8], 67% of bleeding points are arterial, 27% are venous, and 6% show no specific bleeding source, but display diffuse oozing. Abnormal coagulation was thought to have resulted in very mild venous hemorrhage, which would normally be expected to stop spontaneously, and physical movement may have triggered further bleeding, resulting in neck swelling. Third, the drain insertion site is usually covered with blood clots or plasma, but this case showed fresh blood without coagulation when we removed the drain 2 days after reoperation, and the amount of blood collected in the drain bag was only 22 mL. Fourth, subcutaneous hematoma was observed again on POD 11, 5 days after discharge from hospital, despite the patient being discharged after sufficient wound compression and confirmation of resolution of swelling. At this point, we considered the possibility of non-surgical causes of bleeding. Thorough examination then revealed abnormalities in blood coagulability. Because the cause of bleeding had been unclear and a risk of rebleeding was suggested, we had recommended continued hospitalization and observation, but the patient wanted to return home early. Since the pace of bleeding was slow, we had discharged the patient on the premise that we would contact him if any bleeding tendency was identified, but the bleeding recurred 5 days after discharge. The amount of bleeding at this time was about 5 mL, and since no subjective symptoms such as breathing discomfort were present and the bleeding rate was very slow, we judged the possibility of airway symptoms as low. Pressure was applied to the wound with gauze and outpatient follow-up was continued, with the wound absorbed spontaneously. Since then, the patient has not experienced any further neck bleeding.

In this case, no family or medical history was suggestive of hematological disease. Preoperative blood tests were not predictive of abnormalities in coagulation capacity. However, careful elicitation of history during the postoperative interview identified episodes of bleeding difficulty during a previous tooth extraction. If those bleeding episodes had been confirmed, the diagnosis of hemophilia could have been made earlier. Asking patients about previous bleeding episodes preoperatively is recommended, in addition to checking the medical history and blood test results.

## Conclusions

Hemophilia is a risk factor for postoperative bleeding in thyroid surgery. However, mild hemophilia can show normal values for PT and aPTT. We encountered a case of papillary thyroid carcinoma associated with congenital hemophilia A that was only diagnosed after repeated episodes of bleeding.

## Data Availability

The datasets supporting the conclusions of this article are included within the article.

## Abbreviations

Hb: Hemoglobin
PLT: Platelets
PT: Prothrombin time
aPTT: Activated partial thromboplastin time
POD: Postoperative day
